# Chronic Use of Abemaciclib Leading to Breathlessness and Demise: A Case Report

**DOI:** 10.7759/cureus.64774

**Published:** 2024-07-17

**Authors:** Jonathan T Burrows, Rebekah Lantz

**Affiliations:** 1 Internal Medicine, Wright State University Boonshoft School of Medicine, Dayton, USA; 2 Hospital Medicine, Miami Valley Hospital, Dayton, USA

**Keywords:** immune checkpoint inhibitors, intubation, berlin criteria, cancer, er-positive, pd-1 inhibitors, ards, breast cancer, checkpoint inhibitor pneumonitis, abemaciclib

## Abstract

Checkpoint inhibitor pneumonitis (CIP) is a potentially fatal disease that can occur at any duration of treatment. Patients may present with vague respiratory symptoms such as progressive cough, dyspnea, and decreased activity tolerance. Among checkpoint inhibitors, CIP is higher in programmed death 1 (PD-1) inhibitors.

An 82-year-old Latina woman with estrogen receptor (ER)-positive human epidermal growth factor receptor (HER)-2-negative lobular carcinoma of the right breast had been treated by partial mastectomy followed by adjuvant hormonal treatment and radiation in 2014. Then CMF (cyclophosphamide, methotrexate, and 5-fluorouracil) was followed by letrozole and abemaciclib, PD-1, therapy in 2022. In 2023, the patient presented with a dry cough and worsening dyspnea with a new oxygen requirement. She was admitted to the hospital with a diagnosis of multifocal pneumonia and sepsis. She unfortunately developed rapidly higher oxygen requirements and acute respiratory distress syndrome (ARDS) and was ultimately presumed to have CIP. She was intubated on hospital day 6 and extubated on day 12 with no plans for reintubation and do-not-resuscitate status. She subsequently had demise after a period of respiratory arrest.

CIP is rare but associated with fatal outcomes, especially with the development of ARDS. It is important, along the course of cancer treatment and goals of care discussion, to educate patients and their families on possible side effects of chemotherapy and involve specialists early with the goal of lowering mortality rates. Most patients do not survive this unfortunate progression of disease.

## Introduction

As per the American Cancer Society, 1,958,310 incident cases and 609,820 cancer deaths are expected by the end of 2023 [1]. Breast cancer is expected to present 300,590 new times and lead to the demise of 43,700 people within this time [1]. Therefore, early disease detection, medical care, and a treatment plan are of utmost importance.

Breast cancer is generally divided into different categories based on its hormone receptor status for estrogen (ER), progesterone (PR), and human epidermal growth factor-2 (HER-2) receptors [2-3]. ER, PR, and HER-2 status can include one or multiple receptor positivity, which will guide the treatment modality [2-3]. Prevalence for ER-invasive breast cancer is 80%, followed by PR (70%) and HER-2/neu oncogene amplification (20-30%) [3-4]. Triple-negative breast cancers account for 13.2% of all breast cancers [4].

Many factors affect the selection of treatment. The current mainstay involves the surgical removal of the lesion and associated lymph nodes where possible [5]. This can be in the form of either a lumpectomy or a mastectomy. Breast-conserving therapy (BCT) is typically pursued [5]. If unresolved with resection, adjuvant whole breast radiation may be required [5]. In the setting of ER+, adjuvant hormonal therapies with selective estrogen receptor modulators (SERMs) tamoxifen or raloxifene or the aromatase inhibitor (AI) anastrozole are usually implemented [5]. If a patient has a HER-2/neu form of breast cancer, the current recommendation is to add on trastuzumab [5]. There are additional options for biologics, which is a topic currently undergoing rapid expansion with an improved understanding of immune therapy [5].

Immune checkpoint inhibitors (ICIs) have been studied for utility in cancer since the 1990s and became Food and Drug Administration (FDA) approved in 2011 with the agent ipilimumab, an ICI targeting cytotoxic T-lymphocyte-associated protein 4 (CTLA-4) [6-7]. However, ICI use in breast cancer remained scarce until accelerated approval status came for pembrolizumab in PDL-1-positive triple-negative breast cancers in 2020 [8]. Further indications for pembrolizumab were approved in 2021 for "high-risk, early-stage, triple-negative breast cancer (TNBC) in combination with chemotherapy as neoadjuvant treatment, and then continued as a single agent as adjuvant treatment after surgery" [9]. In 2023, abemaciclib was allowed when used in adjuvant therapy with estrogen-modulating endocrine therapies, such as tamoxifen or aromatase inhibitors, for hormone receptor-positive, HER-2-negative, node-positive, early breast cancer that had a high likelihood of recurrence [10].

Approximately 20% of breast cancers become resistant to initial treatment due to pharmacologic resistance, which both worsens the prognosis and requires a new method of treatment [11-13]. Additionally, the cyclin D, cyclin-dependent kinase 4 (D/CDK), retinoblastoma pathway is involved in many cancers and, therefore, remains an important target for new pharmacologic interventions [11]. Abemaciclib, palbociclib, and ribociclib are CDK4/6 inhibitors that target the D/CDK/retinoblastoma pathway, as a downstream result of the ER complex signaling machinery [14].

Despite its advantages in extending mortality by preventing the proliferation of breast cancer cells, variable life-threatening risks and side effects are related to ICI treatment, including pneumonitis and potential downstream acute respiratory distress syndrome (ARDS) [15-16]. This is termed checkpoint inhibitor pneumonitis (CIP) and initially involves dyspnea with or without other respiratory symptoms, coupled with new lesions on chest CT where pulmonary infection and cancerous etiology are excluded [2,14,16].

## Case presentation

The patient is an 85-year-old Latina woman with a past medical history significant for type II diabetes mellitus, diastolic heart failure, gastroesophageal reflux disease (GERD), and hypertension. She also had a history of right-sided breast cancer originally diagnosed in 2014 in Puerto Rico as cT3, N1, M0, but the complete pathology was not known. At that time, she was treated with neoadjuvant therapy, partial mastectomy, followed by radiation and the extended hormonal treatment, exemestane. In 2021, after she had moved to the United States, this was followed with a screening mammogram which was unfortunately positive and considered a recurrence. Based on extension and biopsy results, she was staged as IIIb, T4b, N1aM0 invasive lobular carcinoma. Pathology was weakly ER+, PR(-), and HER-2(-). Treatment entailed right breast-modified radical mastectomy and axillary lymph node dissection. She underwent an uneventful six cycles of CMF (cyclophosphamide, methotrexate, and 5-fluorouracil) therapy. Restaging nuclear medicine positron emission tomography (PET)-computed tomography (CT) showed no active disease, and she was considered in remission. However, due to concern about past recurrence while on an AI, the oncologist placed her on letrozole and abemaciclib.

**Figure 1:**
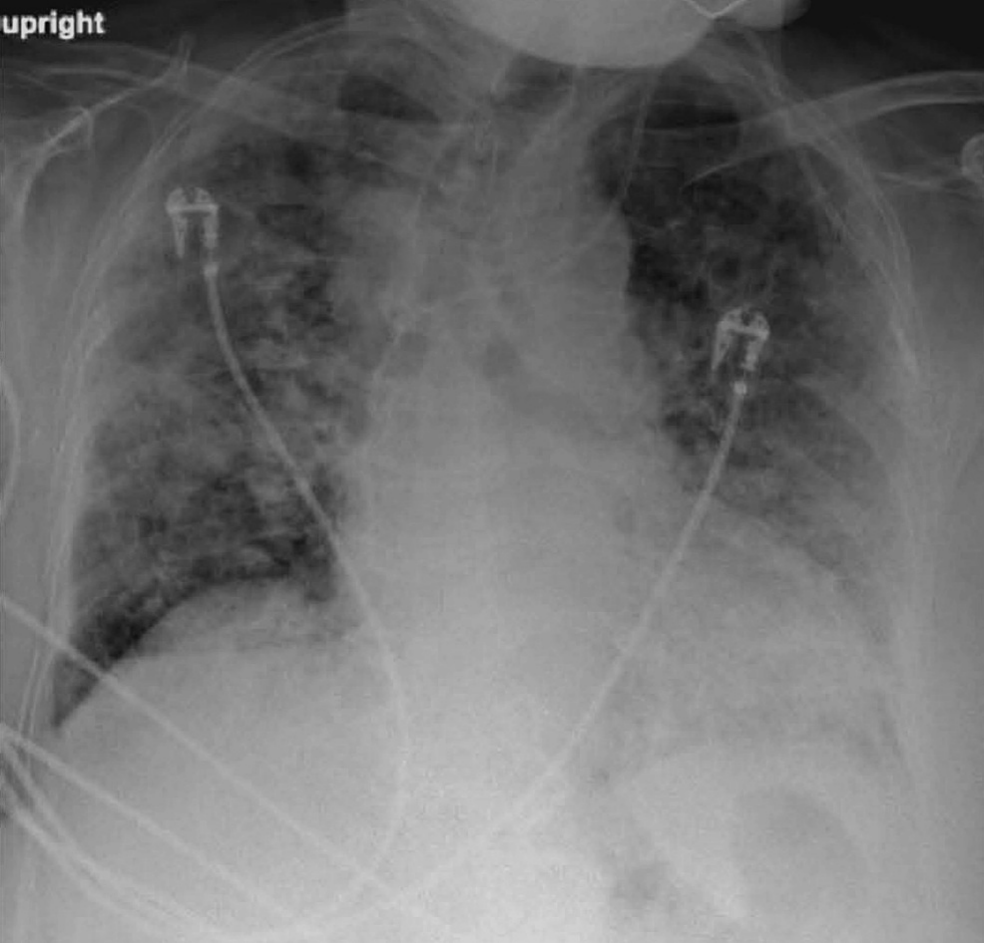
Admission chest X-ray showed bilateral parenchymal opacification

**Figure 2:**
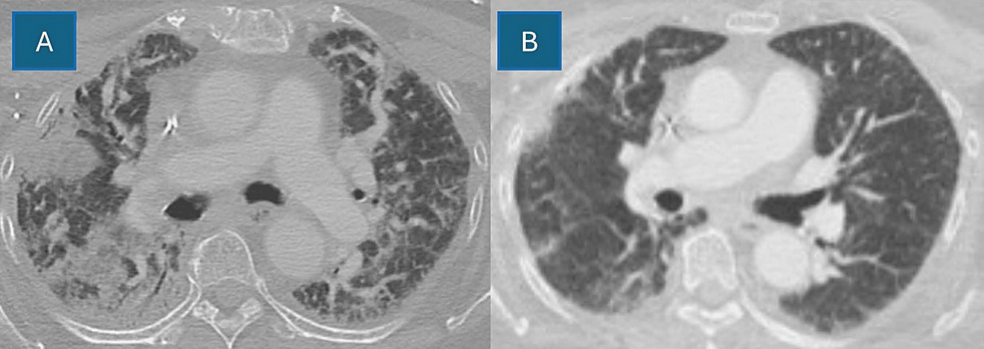
(A) Admission contrasted chest CT demonstrated multifocal airspace disease throughout lung fields, worse on the right. Background subpleural reticulation with underlying interstitial lung disease/fibrosis and diffuse peri-bronchial wall thickening is also present. (B) Comparator chest CT from 2022 with baseline subpleural reticulation and underlying fibrosis

In April 2023, she presented to urgent care complaining of two days of nonproductive cough and progressive shortness of breath. She was afebrile and denied other respiratory symptoms. She only had a two-pack-year history of ever smoking and was not on any maintenance or rescue inhalers. She did not have underlying asthma, or chronic lung disease, and had no home oxygen requirement. Blood pressure was 151/104, pulse 109 beats per minute, respiratory rate 20 respirations per minute, and temperature 98.9°F. Oxygen was 77% on room air, which improved to 93% with 2L by nasal cannula (NC). She was transported to the nearest emergency department (ED) for further evaluation given the hypoxia. In the ED, 4L was required to maintain >92% oxygen saturation. A chest X-ray (CXR) was significant for bilateral parenchymal opacification (Figure 1). A CT showed multifocal airspace disease throughout lung fields, worse on the right. There was background subpleural reticulation with underlying interstitial lung disease or fibrosis and peri-bronchial thickening (Figure 2A). Figure 2B provides a comparator chest CT from the previous year in 2022. Labs were significant for a C-reactive protein of 5.94 mg/dL (reference <0.80 mg/dL) and a mild thrombocytopenia 127 K/uL (reference 140-400 K/uL). Based on her presentation meeting sepsis criteria and suspicion for underlying community-acquired pneumonia, IV ceftriaxone 2 g and 500 mg azithromycin daily were initiated. Due to underlying wheeze, she was also started on dexamethasone 6 mg daily and admitted to the hospital for further workup.

On hospital day 1, she remained at a 2-3L oxygen requirement but began to respiratory decline on day 2 with increased needs to 11L, and pulmonary was consulted. In the evening, oxygen needs further increased to a 15L heated high-flow nasal cannula (HHFNC). She did not tolerate bilevel positive airway pressure (Bi-PAP) due to anxiety.

On day 3, despite being on antibiotics and corticosteroids, there was no marked improvement, so a repeat CXR was imaged and showed further patchy interstitial opacification. Her clinical picture was now indicative of mild ARDS. Her arterial oxygen pressure (PaO2)-to-fraction of inspired oxygen (FiO2) ratio was 204 mmHg based on an arterial blood gas value of PaO2 67.3 mmHg and 11L oxygen requirement (0.11) above 0.21 atmospheric (67.3 mmHg/0.32=204 mmHg). Throughout the day, her oxygen requirement exceeded 70L via HHFNC at 100% FiO2, and she was trialed again on Bi-PAP which was intermittently tolerated through day 5. Anxiety due to high air pressures was of issue, unresolved with careful anxiolytics, hydroxyzine 25 mg. Benzodiazepines were not administered due to concerns about depressing her respiratory drive. Blood cultures resulted as no growth after five days. Viral polymerase chain reaction (PCR) panel including SARS-CoV-2, influenza A and B, and respiratory syncytial virus was not detected. *Legionella* and *Streptococcus pneumoniae* urine antigens were negative assays. Additionally, the procalcitonin level was 0.08 ng/mL (reference 0.00-0.09). Bacterial and viral pneumonia were effectively ruled out. A transthoracic echocardiogram had a normal ejection fraction of 55-60% and normal structure and function of the valves, atria, and ventricles. The right ventricular systolic pressure was 81 mmHg, consistent with severe pulmonary hypertension. Overall, ARDS pneumonitis was the probable etiology, secondary to abemaciclib. Antibiotics were discontinued at this time, and steroids were advanced to methylprednisolone 40 mg twice daily.

On day 6, pulmonary and the family elected to intubate, understanding that she was at high risk of being unable to extubate. This was true when she did not tolerate oxygen weaning in subsequent days. Resuscitation goals were further discussed with the family who elected for a limited code status in which no cardiac resuscitation or medications would be desired. Endotracheal intubation was offered; however, the family elected to avoid further invasive procedures.

On day 11, she appeared to be weaning well on ventilator-positive end-expiratory pressure (PEEP), and a trial of extubation was discussed. The family decided that in the case of extubation failure, reintubation would not be considered and they would enact comfort care measures.

On day 12, she passed a spontaneous breathing trial (SBP) and was extubated to Bi-PAP. She again had anxiety and discomfort that were approached with careful consideration of her breathing status. Effective anxiolytics such as benzodiazepines were considered but not given due to concern for respiratory depression. Ultimately with decision-making capacity, the patient desired to be removed from Bi-PAP, understanding that this would probably result in her demise. She and her family agreed to pursue comfort goals, and she was therefore made do-not-resuscitate comfort care (DNR-CC). She expired on day 13, related to progressive drug-induced interstitial lung disease and as a result of CIP.

## Discussion

Treatments for breast cancer have important side effects, and our case is one of symptomatic CIP. Symptoms can be graded from 1 to 5, where 1 indicates no symptoms and 5 indicates death [17]. There is currently no guideline to define optimal management, but it is generally recommended to stop the offending medication at grade 1 of symptoms and monitor for progression over the next 4-6 weeks [17]. If symptoms or radiographic progression occur, glucocorticoids should be initiated [17]. For grade 2 or higher CIP, high-dose glucocorticoid therapy must be initiated [15]. CIP can unfortunately progress to grade 4, which is classified as a life-threatening dyspnea and ARDS, often necessitating intubation [15].

After infection, pulmonary edema, and other causes of acute hypoxemic respiratory failure have been ruled out, ARDS can be diagnosed [16]. The Berlin criteria are used for diagnosis and severity [18] (Table 1).

**Table 1:** Berlin criteria for ARDS ARDS: acute respiratory distress syndrome; CXR: chest X-ray; CT: computed tomography; FiO2: fraction of inspired oxygen; PaO2: partial pressure of arterial oxygen; PEEP: positive end-expiratory pressure; CPAP: continuous positive airway pressure Referenced from Ferguson et al. [18]

Berlin criteria for ARDS
Respiratory symptom onset occurs within one week of a recognized clinical insult, or there is evidence of new or worsening symptoms over one week.
Bilateral opacities are evident on a CXR or CT. Opacities should not be solely attributed to pleural effusions, lobar collapse, lung collapse, or pulmonary nodules.
The patient's respiratory failure should not be primarily attributed to cardiac failure or fluid overload. An objective assessment, such as echocardiography, is required to rule out hydrostatic pulmonary edema if no risk factors for ARDS are identified.
A significant impairment in oxygenation must be present, as determined by the ratio of arterial oxygen tension to the fraction of inspired oxygen (PaO2/FiO2). The degree of hypoxemia determines the severity of ARDS:
Mild ARDS: The PaO2/FiO2 is >200 mmHg, but ≤300 mmHg, on ventilator settings that include PEEP or CPAP ≥5 cm H2O.
Moderate ARDS: The PaO2/FiO2 is >100 mmHg, but ≤200 mmHg, on ventilator settings that include PEEP ≥5 cm H2O.
Severe ARDS: The PaO2/FiO2 is ≤100 mmHg on ventilator settings that include PEEP ≥5 cm H2O.

In the management of such patients, multiple modalities are often required to maintain adequate oxygenation such as NC, HHFNC, continuous positive airway pressure (CPAP), Bi-PAP, and endotracheal tube (ETT) intubation.

NC is a device that delivers low to moderate concentrations of oxygen via two nasal prongs [19]. It is generally used for patients who require only mild to moderate supplementation of oxygen and is often used at low flow rates such as between 1 and 6 liters per minute (LPM) [19]. NC delivers a continuous flow of oxygen, but the exact inspired oxygen concentration is dependent on the breathing patterns of the individual. HHFNC delivers high-flow, heated, and humidified oxygen by way of special equipment such as a rebreather mask or a venturi mask [19]. HHFNC can deliver flow rates usually between 20 and 60 LPM and can be higher, although further care with CPAP, Bi-PAP, or ETT should be considered at this stage. CPAP is a continuous flow of air and oxygen at a set pressure through either a mask or nasal prongs. This method is advantageous because it can be used with various oxygen concentrations dependent on the patient's needs [20]. It works by providing constant pressure throughout the respiratory cycle which aids in preventing airway collapse [19]. Bi-PAP provides two levels of positive airway pressure: a higher level during inhalation and a lower level during exhalation through a mask [19]. Because Bi-PAP provides both inspiratory and expiratory positive airway pressures, physiological manipulation can assist in both oxygenation and ventilation [19]. Finally, ETT involves the placement of a tube through the mouth, or occasionally the nose, directly into the trachea to secure an airway [20]. Both oxygen and positive pressure ventilation can be provided through the ETT [20]. ETT provides a directed and controlled mechanism for mechanical ventilation and oxygen delivery and, therefore, helps ensure that both oxygenation and ventilation are closely monitored and appropriately managed [19].

ICIs combined with traditional therapies have provided a relatively new avenue for breast cancer therapy, and there are many modalities currently being developed. Vaccine delivery is one of these, with the majority of clinical trials focused on deoxyribonucleic acid (DNA), dendritic cells, and peptide-based therapy [20]. There has not been a significant difference between treatment and placebo groups; however, treatments have been able to be personalized by type of breast cancer [20]. Another potential avenue for treatment is adoptive cell therapies (ACTs) which involves finding and isolating tumor-resident and peripheral blood T cells, modifying and activating them eventually, and returning them back to the patient after cell expansion [20]. The different classifications of ACTs include tumor-infiltrating lymphocyte (TIL), T-cell receptor (TCR), and chimeric antigen receptor T (CAR-T) therapies [20]. CAR-T therapy has success in hematologic malignancies, with some promising results in breast cancer as well [20].

Provider awareness is essential in managing the complications that exist in the context of ICIs for breast cancer. While abemaciclib has efficacy for treatment, it is not without associated risks, as this case demonstrates. Recognizing early symptoms of pneumonitis can expedite care. As oncology research ensues, the aim would be to provide effective amelioration of cancer meanwhile minimizing adverse effects. Age-related studies may be able to differentiate if older patients are at higher risk of ICI than younger comparators. Additionally, it may be of benefit for future studies to identify biomarkers that predispose certain patients to an increased risk of developing pneumonitis. By providing alternatives and decreasing risks for patients, there is great potential to maximize patient care and improve outcomes.

## Conclusions

CIP, including from abemaciclib, is a potential side effect during the targeted treatment of advanced and metastatic breast cancer. Although its occurrence is rare, healthcare providers should be aware of its existence and presentation so that symptoms can be managed promptly. Discontinuation, oxygenation, steroid therapy, and early code status discussions should ensue to improve expectations and outcomes. Future studies should evaluate patients at increased risk including older age at diagnosis and predisposing biomarkers. More alternatives and personalized treatment should be considered so that cancer can be ameliorated without causing a fatal compromise.
